# Aldehyde Trapping by ADX-102 Is Protective against Cigarette Smoke and Alcohol Mediated Lung Cell Injury

**DOI:** 10.3390/biom12030393

**Published:** 2022-03-02

**Authors:** Carmen A. Ochoa, Claire G. Nissen, Deanna D. Mosley, Christopher D. Bauer, Destiny L. Jordan, Kristina L. Bailey, Todd A. Wyatt

**Affiliations:** 1 Department of Internal Medicine, College of Medicine, University of Nebraska Medical Center, Omaha, NE 68198-5910, USA; carmen.ochoa@unmc.edu (C.A.O.); deanna.mosley@unmc.edu (D.D.M.); christopher.bauer@unmc.edu (C.D.B.); djordan@creighton.edu (D.L.J.); kbailey@unmc.edu (K.L.B.); 2 Department of Environmental, Agricultural and Occupational Health, College of Public Health, University of Nebraska Medical Center, Omaha, NE 68198-5910, USA; claire.nissen@unmc.edu; 3 Veterans Affairs Nebraska-Western Iowa Health Care System, Omaha, NE 68105, USA

**Keywords:** alcohol, cigarette, AUD, acetaldehyde, malondialdehyde, lung

## Abstract

Most individuals diagnosed with alcohol use disorders smoke cigarettes. Large concentrations of malondialdehyde and acetaldehyde are found in lungs co-exposed to cigarette smoke and alcohol. Aldehydes directly injure lungs and form aldehyde protein adducts, impacting epithelial functions. Recently, 2-(3-Amino-6-chloroquinolin-2-yl)propan-2-ol (ADX-102) was developed as an aldehyde-trapping drug. We hypothesized that aldehyde-trapping compounds are protective against lung injury derived from cigarette smoke and alcohol co-exposure. To test this hypothesis, we pretreated mouse ciliated tracheal epithelial cells with 0–100 µM of ADX-102 followed by co-exposure to 5% cigarette smoke extract and 50 mM of ethanol. Pretreatment with ADX-102 dose-dependently protected against smoke and alcohol induced cilia-slowing, decreases in bronchial epithelial cell wound repair, decreases in epithelial monolayer resistance, and the formation of MAA adducts. ADX-102 concentrations up to 100 µM showed no cellular toxicity. As protein kinase C (PKC) activation is a known mechanism for slowing cilia and wound repair, we examined the effects of ADX-102 on smoke and alcohol induced PKC epsilon activity. ADX-102 prevented early (3 h) activation and late (24 h) autodownregulation of PKC epsilon in response to smoke and alcohol. These data suggest that reactive aldehydes generated from cigarette smoke and alcohol metabolism may be potential targets for therapeutic intervention to reduce lung injury.

## 1. Introduction

Cigarette smoking is the number one cause of preventable disability and death in the United States, and more than 16 million Americans live with a disease related to cigarette smoking including cancer, heart disease, diabetes, and chronic lung diseases such as COPD and chronic bronchitis [1]. Chronic alcohol use is strongly linked to cigarette smoking. There is a well-established connection between alcohol use and cigarette smoking. More than 80% of individuals who are alcohol-dependent report that they also smoke cigarettes [2]. As the lung injury effects of polysubstance abuse are understudied [3], it is important to consider the combination effects of alcohol and cigarette use to identify the mechanisms of tissue damage that occur.

Alcohol use has an impact on almost every organ in the body, including the lungs. Individuals who drink heavily are at a higher risk of developing a variety of lung pathologies. Chronic alcohol use increases susceptibility to bacterial pneumonia by impairing the ability of alveolar macrophages to perform phagocytosis [4]. Respiratory syncytial virus (RSV) risk is increased in chronic alcohol users by impeding mucociliary clearance, which propels inhaled particles, microbes, toxins, and debris out of the lungs [5]. There is also an increased risk of developing acute respiratory distress syndrome (ARDS) [6].

The majority of alcohol is metabolized in the liver. A small amount is metabolized in the lungs by the alcohol dehydrogenase, CYP2E1. CYP2E1 is especially implicated during chronic alcohol abuse [7] and plays a major role in the oxidant-mediated production of malondialdehyde [8,9]. Volatile aldehydes such as acetaldehyde and malondialdehyde are present in large amounts in cigarette smoke. These aldehydes impair DNA, induce mutagenic adducts, promote lipid peroxidation, and generate reactive oxygen species as well as other free radicals [8,10,11]. The combined impact of alcohol and cigarette smoke has unique effects in the airway [12] by covalently modifying proteins and altering inflammatory responses [13]. Acetaldehyde causes lung injury by forming adducts with proteins. Both acetaldehyde and malondialdehyde are associated with the production of reactive oxygen species, which impair the body’s ability to detoxify reactive oxygen intermediates and products [8], thereby decreasing mucociliary clearance and leaving pathogens and other environmental debris improperly cleared from the airway.

The lungs’ innate defense via mucociliary clearance, which is quantified by cilia beat frequency (CBF), decreases when exposed to cigarette smoke and alcohol in a protein kinase C epsilon (PKCε) dose-dependent manner [14]. Cigarette smoke and alcohol stimulate PKCε and inhibit ciliary motility, leading to the slowing of cilia and eventual ciliated cell detachment. PKCε is transiently activated 3–6 h after consumption to slow cilia beat frequency. By 24 h, PKCε is autodownregulated, leading to deciliation and ciliated cell detachment.

ADX-102 is a novel reactive aldehyde species inhibitor that is currently in development for treating dry eye disease. It covalently binds aldehydes to decrease eye inflammation [15]. Due to the high amounts of reactive aldehydes produced in alcohol metabolism and cigarette smoke, this study aimed to use aldehyde trapping to demonstrate the role of aldehydes in smoke and alcohol mediated bronchial epithelial dysfunction. We hypothesized that ADX-102 prevents the activation of PKC isoform activity, thereby decreasing the amount of damage in the airway caused by alcohol and cigarette smoke.

## 2. Materials and Methods

### 2.1. Cell Culture

BEAS-2B, a human bronchial epithelial cell line, cells were obtained from the American Type Culture Collection (ATCC, Manassas, VA, USA). Cell cultures were grown at 37 °C and 5% CO_2_ in M-199 (Gibco, Thermo Fisher Scientific, Waltham, MA, USA) with 10% fetal bovine serum (FBS) supplemented with penicillin–streptomycin (Gibco, Thermo Fisher Scientific, Waltham, MA, USA) and amphotericin B (Mediatech, Inc., Manassas, VA, USA) in collagen-coated (Invitrogen, Thermo Fisher Scientific, Waltham, MA, USA) tissue culture flasks. A transformed human bronchial epithelial cell line, 16HBE14o-, was obtained from the late D. C. Gruenert (University of California, San Francisco, CA, USA), who cryopreserved low-passage aliquots. Cells were grown in Dulbecco’s Modified Eagle Medium (DMEM) with 10% fetal bovine serum and penicillin–streptomycin supplementation.

For passaging, monolayers were treated with trypsin for 5 min at 37 °C for harvesting followed by 10% FBS to halt the trypsin. Cells were resuspended, counted, and plated at 3 × 10^5^ cells in 800 µL per well in 24 well tissue culture plates coated for a minimum of 10 min with 1% collagen. Cells were left to attach and become 80% confluent. Prior to experimental treatments, cells were washed with PBS (pH 7.4) in fresh medium. Once treatment was added, cells were allowed to incubate at 37 °C and 5% CO_2_ in a standard cell culture incubator (model MCO-19AICUV-PA; Sanyo, Wood Dale, IL, USA). The same incubator was used for all trials.

Using a modified method of that used by Dossou et al. [16], mouse tracheal epithelial cells (MTECs) were cultured using adult wild-type C57BL/6 mice that were euthanized via isoflurane inhalation (Sigma-Aldrich, St. Louis, MO, USA). All uses of mice were reviewed and approved by the Institutional Animal Care and Use Committee of the University of Nebraska Medical Center. Tracheae were dissected and immediately placed in chilled Ham’s F-12 Nutrient medium (Thermo Fisher Scientific, Waltham, MA, USA) containing 1.5 mg/mL pronase (Sigma-Aldrich, St. Louis, MO, USA). The tracheae were left in this solution to dissociate overnight at 4 °C. The dissociated cells were harvested in 0.5 mL of MTEC basic medium containing Dulbecco’s Modified Minimal Essential Medium (DMEM; Invitrogen); F-12 Nutrient mixture, containing glutamine (200 mM/10 mL; Invitrogen), penicillin–streptomycin (10 units, milligrams per milliliter), and gentamycin (50 mg/mL; both purchased from Sigma-Aldrich St. Louis, MO, USA); and amphotericin B (1.25 mg/mL; Sigma-Aldrich). Cells then incubated for 3.5 hours at 37 °C in air with 5% CO_2_ on 35 × 10 mm^2^ Falcon culture dishes (Thermo Fisher Scientific, Waltham, MA, USA). Resuspension of cells was completed in 250 mL of MTEC-supplemented medium made up of MTEC basic medium, fetal bovine serum (Thermo Fisher Scientific, Waltham, MA, USA), insulin (10 mg/mL), transferrin (5 mg/mL), recombinant human epidermal growth factor (5 mg/mL), bovine pituitary extract (Lonza, Rockland, ME, USA), cholera toxin (62 mg/mL), and retinoic acid (5%; Sigma-Aldrich St. Louis, MO, USA). Cells were plated at 1 × 10^5^ cells/mm^2^ on 6.5 mm diameter Costar membrane inserts (Corning Life Sciences, Tewksbury, MA, USA); coated with rat tail collagen, type I (Becton Dickinson, Bedford, MA, USA); and allowed to incubate at 37 °C in air supplemented with 5% CO_2_. MTEC-supplemented medium was removed and replaced after 3 days of incubation. When the cultures were confluent (5–6 days), an air–liquid interface (ALI) was established. For ALI, medium was removed from the apical portion of the membrane, resulting in the exposure of the cultures to air on the apical surface and medium on the basal surface. The medium was replaced every 2–3 days until fully formed cilia appeared and exhibited a baseline 10–11 Hz cilia beat frequency.

### 2.2. Cell Treatments

BEAS-2B cells were treated with 1–100 µM ADX-102 diluted in M-199 medium containing 10% fetal calf serum for up to 24 h in the presence or absence of 5% cigarette smoke extract (CSE). Ciliated MTECs were pretreated with 10 nM to 100 µM ADX-102 for 1 h, followed by the addition of 50 mM ethanol and 5% CSE for up to 24 h. 16HBE cells were treated with either 50 mM ethanol, 5% CSE, or both ethanol and CSE in the presence or absence of 10 µM ADX-102 for up to 72 h.

### 2.3. Cigarette Smoke Extract Preparation

Cigarette smoke extract (CSE) was prepared fresh each day using reference cigarettes (85 mm, filtered, 1R6F) that were obtained from the Center for Tobacco Reference Products (CTRP), a division of the Kentucky Tobacco Research & Development Center at the University of Kentucky (Lexington, KY, USA). Cigarettes were smoked by connecting a cigarette to a peristaltic pump (model ATS-P, Bentley Laboratories, Santa Anna, CA, USA). It was lit and bubbled through 20 mL of sterile phosphate-buffered saline (PBS, pH 7.4) and equilibrated at a burn rate of 6 min, or 160 cm^3^/min. The discharge end of the tube was inserted into the medium in a 50 mL conical tube, and parafilm was wrapped around the top of it to prevent smoke from escaping. Once approximately 60–75 mm of the cigarette was burned, the pump was turned off and the medium tube was capped as 100% CSE. Undiluted CSE medium was sterile-filtered (0.22 µm) and diluted into sterile culture medium to a final concentration of 5% by volume for maximal viability, as reported in [17]. All CSE was used within 12 h of preparation unless overnight pretreatment was indicated.

### 2.4. In Vitro Wound Closure (Migration) Assay

BEAS-2B cells were grown to confluency in a flat-bottomed, 24-well or 60 mm tissue culture dish. A circular “wound”, approximately 1 mm^2^, was created within the cell monolayer by a sterile scraper. Differences in rate of wound closure between 60 mm dishes and 24-well plates were accounted for by adjusting the volume as a ratio of volume to surface area, which created an equipotential rate in wound closure, as described in [18].

Migration was monitored with a phase-contrast microscope with a video camera attachment (Olympus, Center Valley, PA, USA). Camera output was captured with image analysis software (NIH ImageJ v1.45 for Mac OS X, Bethesda, MD, USA). Each wound was individually photographed; its images were analyzed at specific time points, and the area of the wound was measured. Dishes remained in the incubator between measurements. The wound area was reduced as cells migrated into it. BEAS-2B cells were grown on 60 mm tissue culture dishes to confluency for kinase experiments. Using a sterile “cell rake”, cell monolayers were wounded, removing cells in a grid-like pattern [19], which removed approximately 14.1% of total cells. Linear wounds averaged 0.3 mm in width, 2 mm apart. 

### 2.5. PKCα and PKCε Activity Assays

The conditioned medium from treated cells was removed. Cells were flash-frozen with liquid nitrogen in a cell lysis buffer, as described in [20]. Cells were thawed and removed from the plate with a cell lifter. They were sonicated and centrifuged at 10,000× *g* for 30 min at 4 °C. The soluble cytosolic fraction was collected; the remaining cell pellet was resuspended in cell lysis buffer containing 0.01% Triton X-100 (Sigma-Aldrich, St. Louis, MO, USA), and the particulate fraction was sonicated again. The isoform activity of PKC was determined in crude lysates by the method previously described in [14]. A reaction mix of 24 mg/mL PMA, 30 mM dithiotreitol, 150 mM ATP, 45 mM Mg-acetate, PKC isoform-specific substrate peptide (Enzo Life Sciences, Farmington, NY, USA), and 10 mCi/mL [γ-^32^P]-ATP (Perkin Elmer, Waltham, MA, USA) was mixed in a Tris–HCl buffer (pH 7.5) to measure specific PKC isoform activity. Aliquots (20 µL) of cytosolic or particulate fraction were chilled at 4 °C and added to 40 mL of the reaction mix and incubated for 15 min at 30 °C. To halt incubation, a 60 µL sample was spotted onto P-81 phosphocellulose paper (Jon S. Oakhill, St. Vincent’s Institute of Medical Research, Fitzroy, Australia); then, it was washed 5 times in 75 mM phosphoric acid for 5 min, washed in 100% ethanol for 1 min, dried, and counted in nonaqueous scintillant (National Diagnostics, Atlanta, GA, USA). PKC activity was expressed in relation to the total amount of cellular protein assayed as picomoles of phosphate incorporated per minute per milligram.

### 2.6. Cilia Beating Assay (SAVA)

Sisson et al. [21] provided an in-depth description and characterization of the Sisson-Ammons Video Analysis (SAVA) system. This device is used to measure ciliary beat frequency (CBF) of actively beating ciliated cells at a given time point. Motility is quantified by whole-field analysis, which is a combination of phase-contrast microscopy and computerized frequency spectrum analysis. Number of motile points for each 3 s digital video was determined by a software algorithm in SAVA that assesses the change in light intensity for each 16-pixel zone represented by a 4 × 4-pixel area. For every 640 × 480-pixel video image, the number of motile zones was calculated from 19,200 possible total zones. Motile points decreased over time as cilia stopped beating or detached from the monolayer.

### 2.7. Transepithelial Resistance Assay

Electric cell–substrate impedance sensing (ECIS, Applied BioPhysics, Troy, NY, USA) was used to determine cell transepithelial electrical resistance (TEER). The 16HBE cell line was used as this cell line produces the greatest barrier resistance in submerged culture monolayers. Cells were grown on collagen-coated 8W1E PET arrays and 5 × 10^5^ cells were plated into each well. They were grown to confluency and treated with different doses of cigarette smoke or ethanol in the presence or absence of ADX-102 (TargetMol, Wellesley Hills, MA, USA) for a maximum of 72 h. Controls consisted of medium-only wells with and without cells. Resistance of cell barriers was measured on the basis of changes in resistance and capacitance to current flow applied to the electrode arrays at 4000 Hz. To test for differences in TEER, resistance values were obtained every 15 min. Baseline was established by comparing changes in resistance over time to wells without either ethanol, cigarette smoke, or a combination of the two with and without ADX-102. Background resistance values for a well containing only medium without the presence of a cell monolayer were subtracted.

### 2.8. Cell Viability Assay

A conditioned medium (50 µL) was assayed for viability of cells using the provided instructions of a commercially available kit (MAK066-1KT, Sigma-Aldrich, St. Louis, MO, USA) to measure lactate dehydrogenase (LDH) release. Lysed cells were used as a positive control for LDH release. Neither CSE nor alcohol at the concentrations and exposure times used in this study cause cell death [13,14,17,22].

### 2.9. ELISA for MAA Adducts

MAA-adducted proteins were assayed in human BAL samples by indirect competitive ELISA as previously described in [13].

### 2.10. Statistical Analysis

All experiments were performed in triplicate. Each data point represented the mean of a minimum of three independent measurements. Each data point graphically presented in this article represents at least nine measurements used to generate the standard error of the mean. Data were analyzed using Graph Pad Prism (v9.2.0 for Mac, GraphPad Software, San Diego, CA, USA). Data are represented in this paper as mean ± standard error. Data were analyzed for statistical significance using a two-way ANOVA using Bonferroni or Tukey’s multiple comparison posttest corrections, depending on equality of sample sizes between repeated experiments. Significance was accepted at a 95% confidence interval.

## 3. Results

### 3.1. Only High Concentrations of ADX-102 Affect Cell Viability

**Figure 1:**
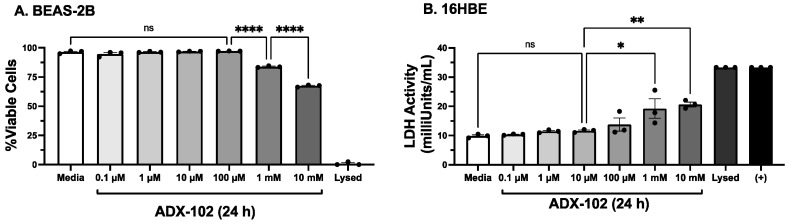
ADX-102 and bronchial epithelial cell viability. Medium release of lactate dehydrogenase (LDH) was measured and expressed as % viability in BEAS-2B cells (**A**) or LDH activity in 16HBE cells (**B**) after 24 h treatment with 0.1 µM to 10 mM ADX-102 in M-199 with 10% serum (Media). An equal number of cells were sonicated for maximum LDH release (Lysed). Positive assay control (+) was provided by a manufacturer. * *p* < 0.05 and ** *p* < 0.01 vs. 10 µM ADX-102; **** *p* < 0.0001 vs. 0–100 µM ADX-102. Bars represent SEM of biological *n* of three with three technical replicates. ns = not significant.

ADX-102 use on airway epithelial cells has not been previously reported. To control against any artifacts in cell responses due to toxicity, we treated the bronchial epithelial cell line, BEAS-2B, with various concentrations (up to 10 mM) of ADX-102 for 24 h in culture. Then, we collected medium supernates and assayed them for LDH release. No change in the viability (≥95%) of BEAS-2B cells was observed over this 24 h period after adding concentrations up to and including 100 µM of ADX-102 (Figure 1A). However, a significant decrease in viability was detected in BEAS-2B cells similarly treated with 1 mM (≤84%) or 10 mM (≤67%) of ADX-102. Likewise, a significant release of LDH was detected in supernates of 16HBE cells exposed to 1–10 mM of ADX-102, and there was no detectable increase in LDH observed at or below 100 µM (Figure 1B). Treatment up to 72 h with >100 µM of ADX-102 resulted in no loss in viability. These same results were observed in primary ciliated MTECs (data not shown). No loss of viability due to 5% cigarette smoke and 50 mM of alcohol treatment (±≤100 µM ADX-102) was detected in any cell type used.

### 3.2. ADX-102 and Wound Repair

**Figure 2:**
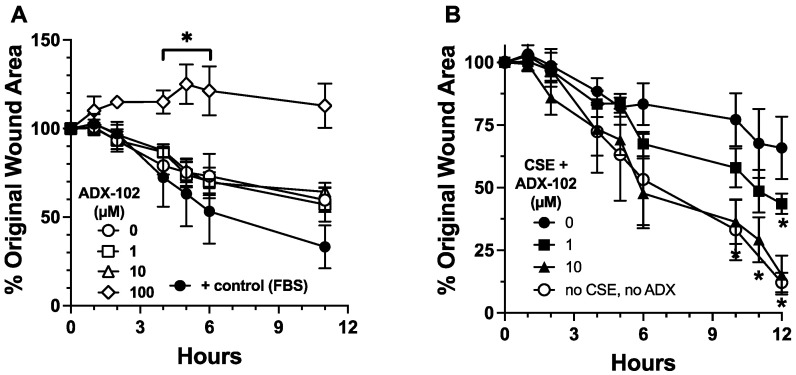
ADX-102 and bronchial epithelial cell migration. Circular wounds in monolayers of BEAS-2B cells were measured for cell migration into the wound area in the presence of 0–100 µM ADX-102 over time (**A**). Positive control for wound closure was M-199 with 10% serum (FBS). In panel (**B**), BEAS-2B cells were wounded in the presence of 5% cigarette smoke extract (CSE) and in the presence of 0–100 µM ADX-102, and % wound closure was measured. * *p* < 0.05 vs. 0 µM ADX-102. Bars represent SEM of *n* = 5, each with three replicates.

**Figure 3:**
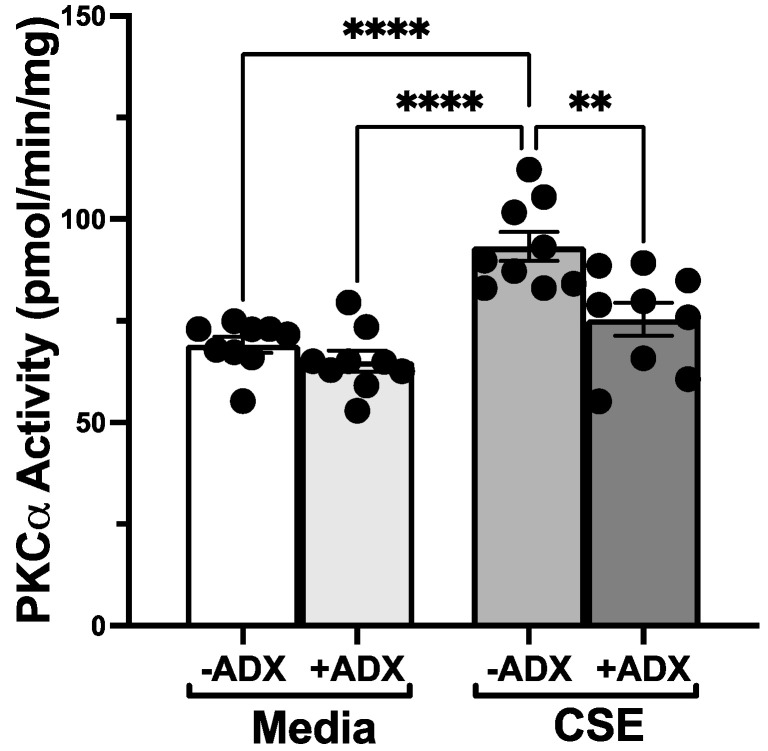
ADX-102 and bronchial epithelial cell protein kinase C alpha (PKCα) activity. BEAS-2B cells were pretreated with or without 10 µM ADX-102 for 1 h prior to treatment with M-199 containing 10% serum (Media) or 5% cigarette smoke extract (CSE) for 1 h. In the absence of ADX-102, **** *p* < 0.0001 CSE vs. Media. In the presence of CSE, ** *p* < 0.01. No ADX vs. ADX. Bars represent SEM of *n* = 9, each with three replicates.

Cigarette smoke impairs the migration of epithelial cells into a wounded monolayer [17]. To examine whether aldehyde trapping impacts the effect of cigarette smoke on epithelial migration, we first determined whether ADX-102 alone alters the migration response of BEAS-2B cells to close a monolayer wound. Compared to a control medium, no change was observed in cell migration in the presence of up to 10 µM of ADX-102 (Figure 2A). However, 100 µM of ADX-102 resulted in a significant slowing of migration as early as 4 h after treatment in culture after the monolayer was wounded. In the presence of 5% CSE, a 1 h pretreatment with ADX-102 reversed CSE-induced slowing of cell migration into a wound in a dose-dependent manner, and there was a significant difference that occurred with 10 µM of ADX-102 (Figure 2B). This response continued for up to 24 h, which was when complete wound closure occurred (10% serum positive control; data not shown). As smoke activation of PKCα is a mechanism for the slowing of epithelial wound repair, and acetaldehyde is a required component of cigarette smoke for PKC activation [22], we examined the effect of ADX-102 on CSE-stimulated PKCα activity in BEAS-2B cells. Treatment for 1 h with 10 µM of ADX-102 alone resulted in no change in baseline PKCα activity (Figure 3). CSE (5%) significantly stimulated PKCα activity by 1 h. However, a 1 h pretreatment with 10 µM of ADX-102 resulted in a significant decrease in CSE-stimulated PKCα activation.

### 3.3. ADX-102 and Cilia Beating

**Figure 4:**
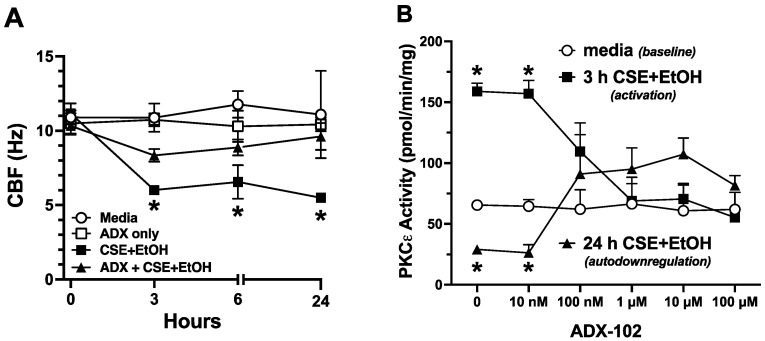
ADX-102 and tracheal epithelial ciliated cell protein kinase C epsilon mediated cilia beating. Ciliated MTECs were treated with or without the combination of 5% cigarette smoke extract (CSE) and 50 mM ethanol (EtOH) in the presence or absence of 10 µM ADX-102, and cilia beat frequency (CBF) was measured from 0–24 h (**A**). Baseline control consisted of DMEM with 10% serum (Media). * *p* < 0.05 ADX vs. no ADX in the presence of CSE + EtOH at 3, 6, and 24 h. Dose–response (0–100 µM) for ADX-102 on combined CSE and EtOH stimulated (3 h) and autodownregulated (24 h) protein kinase C epsilon (PKCε). * *p* < 0.01 vs. medium control. Bars represent SEM of *n* = 6, each with three replicates (**B**).

Cigarette smoke and alcohol co-exposure results in a PKCε-mediated slowing of cilia [14]. To determine if aldehyde trapping impacts this cilia-slowing effect, primary MTECs cultured by air–liquid interface were pretreated for 1 h with 10 µM of ADX-102 prior to combined exposure to 5% CSE and 50 mM of ethyl alcohol (a biologically relevant and nontoxic dose [14]). Then, CBF was measured. MTECs treated with smoke and alcohol showed significantly decreased CBF (from 11 Hz to 6 Hz) by 3 h posttreatment, and this decrease was sustained for up to 24 h (Figure 4A). In the presence of 10 µM of ADX-102, a smoke and alcohol mediated decrease in CBF occurred, but it was less pronounced (from 11 Hz to 8 Hz) and the same as that seen in a control medium. By 24 h, smoke and alcohol had no slowing effect on cilia when ADX-102 was present. In the presence of a control medium, CBF was maintained at approximately 11 Hz for up to 24 h. Concentrations of ADX-102 at or below 1 mM produced no significant change from the baseline CBF. Smoke and alcohol rapidly (3 h) activate PKCε, leading to cilia-slowing. Then, PKCε autodownregulates from 6 to 24 h, resulting in kinase activity levels below that of baseline. In the presence of ADX-102 (1–100 µM), neither the early (3 h) activation nor late (24 h) autodownregulation of PKCε were observed in response to smoke and alcohol (Figure 4B).

### 3.4. ADX-102 and Cilia Loss

**Figure 5:**
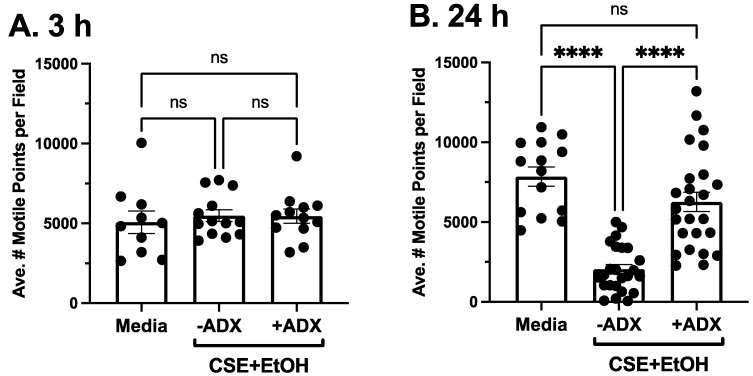
ADX-102 and loss of tracheal epithelial cell cilia. Ciliated MTECs were treated with a combination of 5% cigarette smoke extract (CSE) and 50 mM ethanol (EtOH) in the presence or absence of 10 µM ADX-102, and the average number of motile cilia were measured at 3 (**A**) and 24 h (**B**). **** *p* < 0.01 vs. medium control or presence of ADX. Bars represent SEM of at least *n* = 9 individual experiments. ns = not significant.

PKCε autodownregulation results in cilia loss and ciliated cell detachment [14]. As we found that ADX-102 prevented smoke and alcohol mediated PKCε autodownregulation, we measured the ability of aldehyde trapping to prevent smoke and alcohol induced losses in cilia motility. Using whole-field analysis to capture all the motile points in a complete field, no loss of cilia motile points was observed at 3 h after treatment with smoke and alcohol (when PKCε was catalytically active) (Figure 5A). The average number of moving cilia was similar in both the presence and absence of 10 µM of ADX-102 at 3 h posttreatment. However, by 24 h posttreatment, PKCε was autodownregulated (Figure 4B) and a significant loss in average motile cilia per field was observed (Figure 5B). Cotreatment with 10 µM of ADX-102 prevented this loss in motile cilia. Consistent with cell viability data, concentrations of ADX-102 greater than 1 mM resulted in significant cilia loss and near-complete ciliated cell detachment when 10 mM of ADX-102 was added (data not shown).

### 3.5. ADX-102 and Barrier Function

**Figure 6:**
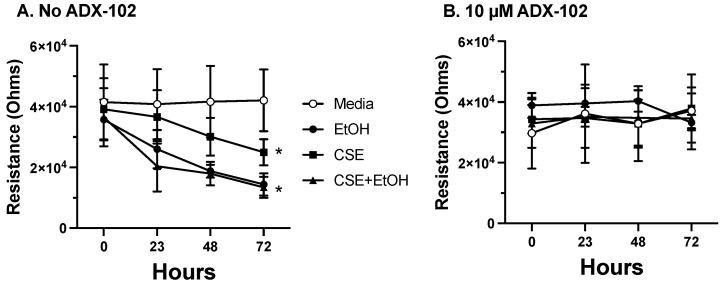
ADX-102 and bronchial epithelial cell permeability. 16HBE cells were grown to confluence until maximal barrier function (Resistance) achieved. Cells were treated with either DMEM and 10% serum (Media), 5% cigarette smoke extract (CSE), 50 mM alcohol (EtOH), or the combination of smoke and alcohol for up to 72 h in the absence (**A**) or presence (**B**) of 10 µM ADX-102. * *p* < 0.02 vs. media at 72 h. Bars represent SEM of *n* = 5, each with three replicates.

Epithelial cell barrier function is negatively impacted by both cigarette smoke [23] and alcohol [24]. Resistance across a confluent monolayer of 16HBE cells was significantly decreased by the addition of 5% CSE at 72 h (Figure 6A). However, treatment with 50 mM of alcohol or a combination of alcohol and CSE decreased the time of barrier permeability to ~24 h. A 10 µM pretreatment with ADX-102 for 1 h prior to alcohol or CSE exposure prevented this loss of resistance and protected barrier function (Figure 6B). Concentrations of ADX-102 up to 1 mM had no effect on resistance (data not shown).

### 3.6. ADX-102 and MAA Adduct Formation

**Figure 7:**
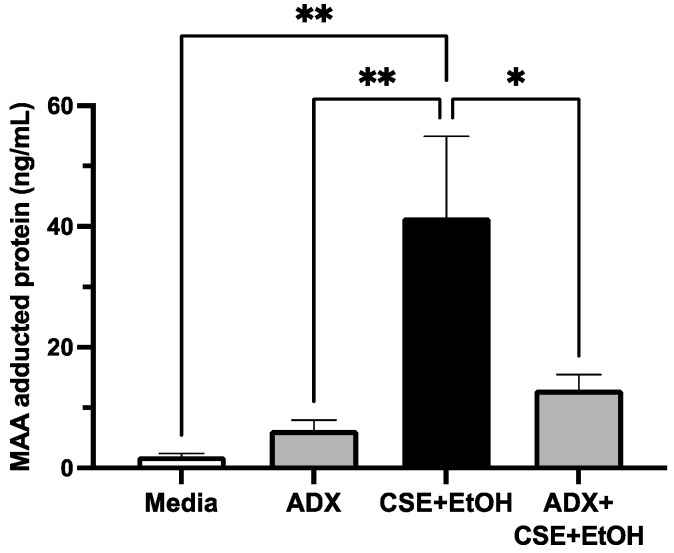
ADX-102 and MAA adduct formation. 16HBE cells were grown to confluence in 60 mm culture dishes. Cells were treated with either M-199+10% serum (Media) or a combination of 5% cigarette smoke extract (CSE) and 50 mM alcohol (EtOH) for 72 h in the absence or presence of 10 µM ADX-102. * *p* <0.04 and ** *p* < 0.003 at 72 h. Bars represent SEM of *n* = 6.

The co-exposure to cigarette smoke and alcohol generates concentrations of malondialdehyde and acetaldehyde that are required for MAA-adducted protein formation in the lungs [24]. To determine if aldehyde trapping prevents smoke and alcohol induced formation of MAA-adducted protein, 16HBE cells were treated with or without 5% CSE and 50 mM of alcohol in the presence or absence of 10 µM of ADX for 72 h. This combination of CSE and alcohol produced small but significant amounts of MAA-adducted protein (Figure 7). However, in the presence of ADX-102, MAA was significantly decreased in cells treated with CSE and alcohol. No significant amounts of MAA-adducted protein were observed using a control medium or ADX-102 alone. 

## 4. Discussion

**Figure 8:**
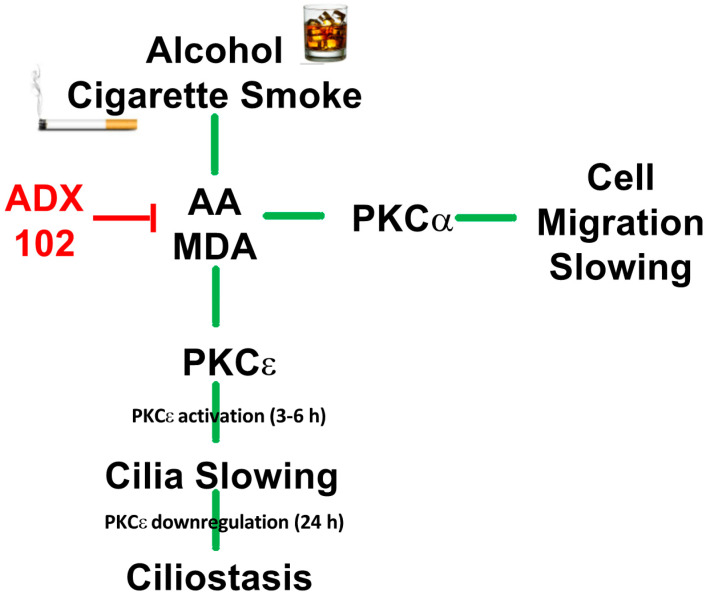
Model diagram for ADX-102 aldehyde-trapping action on cigarette smoke and alcohol mediated injury to airway epithelial cell function.

The lungs are a particularly important target for reactive aldehyde injury in the context of alcohol misuse and cigarette smoking. Alcohol is metabolized into acetaldehyde by alcohol dehydrogenase, and the pyrolysis of tobacco releases large concentrations of acetaldehyde that are directly inhaled by smokers [25]. Lung inflammation induced by reactive oxygen species (ROS) in cigarette smoke produces significant amounts of lipid peroxidation and the formation of malondialdehyde [26]. Similarly, ROS are generated by the metabolism of alcohol through the action of CYP2E1 in the lungs [27]. Acetaldehyde activates PKCα and impairs epithelial cell migration, an important function in re-epithelialization in response to repairing a wound [28]. Malondialdehyde and acetaldehyde covalently bind to lysine residues of target proteins via a Schiff base reaction to form stable protein adducts known as MAA adducts [29]. MAA-adducted proteins bind to airway epithelial cells through the CD204 receptor and activate PKCε [30]. Agents that activate PKCε slow cilia and lead to cilia loss or ciliated cell detachment [14]. We showed for the first time that trapping aldehydes with ADX-102 decreases aldehyde-mediated airway epithelial cell injury (Figure 8).

This study is limited to in vitro observations in airway epithelial cell function. However, these lung cell functions are established models of environmental injury and repair. We utilized three cell types in this study. BEAS-2B cells are optimal for migration studies due to their short passage time but cannot generate cilia for CBF studies and do not form optimal tight junctions for barrier studies. 16HBE cells form excellent tight junctions for barrier studies but do not generate cilia for CBF studies. Primary mouse tracheal epithelial cells can only be cultured in small amounts and grow slowly, but they produce the cilia required for functional studies of CBF. By using the inbred mouse model in this study, human donor variability in the known differential ciliary responses due to environmental exposures, pre-existing lung disease, or age was avoided. Cilia are highly regulable and respond to environmental challenges by increasing their beat frequency to facilitate the effective clearance of particles, microbes, and other pathogens trapped in mucus. However, some agents produce ciliotoxic effects, resulting in a slower cilia beat as well as ciliocytophthoria [31]. Likewise, the impact of a toxin on the maintenance of lung barrier function can be assessed in vitro through measuring resistance across a cell monolayer as a model for potential edematous injury. The repair of an injured epithelial monolayer is governed by the ability of cells to migrate efficiently and rapidly into a wound to facilitate re-epithelialization and the restoration of homeostasis. In each of these models, reactive aldehydes produce such injury through their activation of PKC [32]. We found that ADX-102 inhibits cigarette-smoke-induced delays in cell migration at a lower concentration (10 µM) than that required to prevent the slowing of cilia (100 µM). This may represent differences in aldehyde activation of the two PKC isoforms that govern these cell functions. In addition, we observed that ADX-102 inhibits smoke and alcohol stimulation of PKCε> at much lower concentrations than those required to prevent the slowing of cilia. This is possibly related to different subcellular populations of PKCε in the cell: cilia-localized and cytoplasmic-located kinases [33]. As our catalytic activity assays measured total cellular PKCε, the enzyme specifically dedicated to cilia may require a higher ADX-102 concentration for targeting.

Excessive alcohol use is a public health problem that has been defined and measured using a variety of metrics across different cultures and organizations. According to the National Institute on Alcohol Abuse and Alcoholism (NIAAA), Alcohol Use Disorder (AUD) is an individual’s inability to stop drinking alcohol, despite negative effects, socially, occupationally, or medically [34]. It is measured quantitatively with the Alcohol Use Disorders Identification Test (AUDIT), which is a 10-item screening tool that measures levels of alcohol-related problems [35]. According to the National Survey on Drug Use and Health (NSDUH), in 2019, 14.5 million individuals aged 12 years or older had AUD [36]. The public health burden of alcohol misuse includes the established lung diseases of pneumococcal pneumonia [37], chronic obstructive pulmonary disease [38], and COVID-19 [39]. While the established role of alcohol in lung disease is underappreciated, our current understanding of ROS in environmental injury has the potential to open the door to new and innovative clinical therapeutic interventions.

One novel therapeutic approach is the recent development of aldehyde-trapping drugs. The novel reactive aldehyde species (RASP) inhibitor ADX-102, also known as Reproxalap, was recently reported to be safe and effective for the treatment of dry eye disease (DED) [40,41] as well as ocular inflammatory conditions such as noninfectious anterior uveitis and allergic conjunctivitis [15]. Such clinical trials reported that ADX-102 is safe and effective for human use. Although these studies involved topical treatment, delivery using intraperitoneal injection in mice with resulting anti-inflammatory effects was previously reported [42]. Our study represents the first application of an aldehyde-sequestering compound to address reactive-aldehyde-mediated injury to lung epithelium in vitro. A major limitation of our study is that in vitro cell studies may not translate into the chronic exposure conditions and multifactorial complexities of the in vivo state. As our murine cigarette smoke and alcohol co-exposure model is established [20], translating the findings reported here to an in vivo model is essential to determine whether ADX-102 is useful in protecting against reactive aldehyde lung injury. Further research is required to define any practical implications for ADX-102 as a therapeutic drug for the lungs.

## Data Availability

The data presented in this manuscript are available upon request from the corresponding author.
